# Beyond Self-Report: Tools to Compare Estimated and Real-World Smartphone Use

**DOI:** 10.1371/journal.pone.0139004

**Published:** 2015-10-28

**Authors:** Sally Andrews, David A. Ellis, Heather Shaw, Lukasz Piwek

**Affiliations:** 1 Division of Psychology, Nottingham Trent University, Nottingham, United Kingdom; 2 Department of Psychology, Lancaster University, Lancaster, United Kingdom; 3 School of Psychology, University of Lincoln, Lincoln, United Kingdom; 4 Faculty of Business and Law, University of the West of England, Bristol, United Kingdom; Universitat Wien, AUSTRIA

## Abstract

Psychologists typically rely on self-report data when quantifying mobile phone usage, despite little evidence of its validity. In this paper we explore the accuracy of using self-reported estimates when compared with actual smartphone use. We also include source code to process and visualise these data. We compared 23 participants’ actual smartphone use over a two-week period with self-reported estimates and the Mobile Phone Problem Use Scale. Our results indicate that estimated time spent using a smartphone may be an adequate measure of use, unless a greater resolution of data are required. Estimates concerning the number of times an individual used their phone across a typical day did not correlate with actual smartphone use. Neither estimated duration nor number of uses correlated with the Mobile Phone Problem Use Scale. We conclude that estimated smartphone use should be interpreted with caution in psychological research.

## Introduction

Around 2 billion people use smartphones across the globe, with over half the population in developed countries relying on them daily [1]. This ubiquity means that there is the potential for objective smartphone data to be used to address research questions in the real world [2]. Indeed, there has been a rapid increase in the number of publications examining the relationship between smartphone use, personality, cognition, health, and behaviour e.g. [3–8]. Despite this, smartphones themselves have yet to become a standard item in the psychologist’s research toolbox, and little is known about the validity of self-reported estimates of smartphone use.

Miller recently [9] highlighted how important it is for social science researchers to be current with new developments in smartphone research methods. Perhaps the biggest barrier to exploring the objective (actual) use of smartphone data includes developing suitable apps and the appropriate tools for processing, analysing and visualising big-data sets [10]. Whereas open source software to create *Android* apps is freely available for those with no programming experience [11], there remains no open source software for analysing and visualising the resulting data.

While self-report data can be collected successfully in situations where it is difficult to obtain objective data, this may not be an appropriate measure when it comes to estimating smartphone use. It remains possible that estimates are sufficient for some research questions. but much of the cognitive literature on time-perception suggests we are poor at estimating such durations [12]. Any subjective estimate is also likely to ignore rapid, yet pervasive, checking behaviours [13].

Here we propose that a simple measure—recording when the phone is in use—can provide a vast array of information about an individual's daily routine. We describe and explore different metrics for objective evaluation of smartphone data, and what this can reveal about smartphone use. We include source code for processing, visualising and analysing objective smartphone data, which can be used by those with little to no programming knowledge. As an applied example, we then explore the claim that people engage in habitual smartphone checking behaviours, by correlating self-report smartphone use estimates with actual smartphone use and standardised measures of problem mobile phone use [14]. We finally consider other research questions that could be explored with this methodology.

## Method

### Participants

Twenty-nine participants were recruited (17 female, mean age = 22.52, range = 18–33). All participants owned *Android* smartphones and consisted of staff and students at the University of Lincoln. A priori calculations suggest this number to be adequate for finding a moderate correlation between actual and self-reported use, so we stopped collecting after this number was reached. The study conformed to the recommendations of the Declaration of Helsinki. All participants provided written and oral informed consent after being advised of the purpose of the study, and the type of data being collected. Approval for the project was obtained from the School of Psychology Research Ethics Committee at the University of Lincoln. All participants were reimbursed a small fee (£10) for their time. Two participants were excluded as they had technological problems partway through the study, while four additional participants were excluded from the analysis for not providing all self-report estimates.

### Materials

Smartphone Application: We developed an *Android* smartphone app using *Funf in a Box* [11]. Apps collecting data from *Android* devices are generated by selecting sensors, and specifying sampling frequency. We selected the screen on/off option, resulting in a small app that records a timestamp when a use starts and ends. Data is encrypted and uploaded to a server over Wi-Fi (for more details see [11]). Our app simply recorded a timestamp when the phone became active, and a second when this interaction ended (typically screen use, although this also includes processor intensive activities including calls and playing music).

Mobile Phone Problem Use Scale (MPPUS): This questionnaire consists of 27 items, which have previously demonstrated positive correlations with self-reported mobile phone use [14]. The MPPUS remains a highly cited scale across health and psychological research [15–19], and has been used as an additional means of measuring mobile phone use more generally [6, 20, 21]) (Cronbach's alpha = .89 for standardised items in our sample).

### Procedure

On arrival at the lab, a smartphone application was installed on participants' smartphones. They were then sent a standardised SMS that they were asked to relay back to the experimenter, to determine the length of time taken to check a message. Time taken was recorded from the notification tone until the message had been relayed. Participants were asked to record an estimate each evening of how long they used their phone that day, for the next 14 consecutive days. We asked participants to only estimate their phone use during periods where their phone screen was switched on, as the *Funf* on-off sensor was advertised as measuring screen state. However, during testing, it was discovered that the on-off sensor actually measured whether the phone was in an interactive state, which included activities such as phone calls and listening to music, commonly done with the screen switched off. While we did not analyse the diary data further, it is possible that the process influenced participants’ later estimations of their phone use. When participants returned to the lab after 14 days, they were asked to estimate how much they used their phone on average each day (including calls and listening to music). This measure was used in subsequent analyses of subjective estimates. They were then asked to estimate how many times they use their phone each day (number of uses), and finally were asked to complete the MPPUS. The app was then uninstalled from their device.

**Fig 1:**
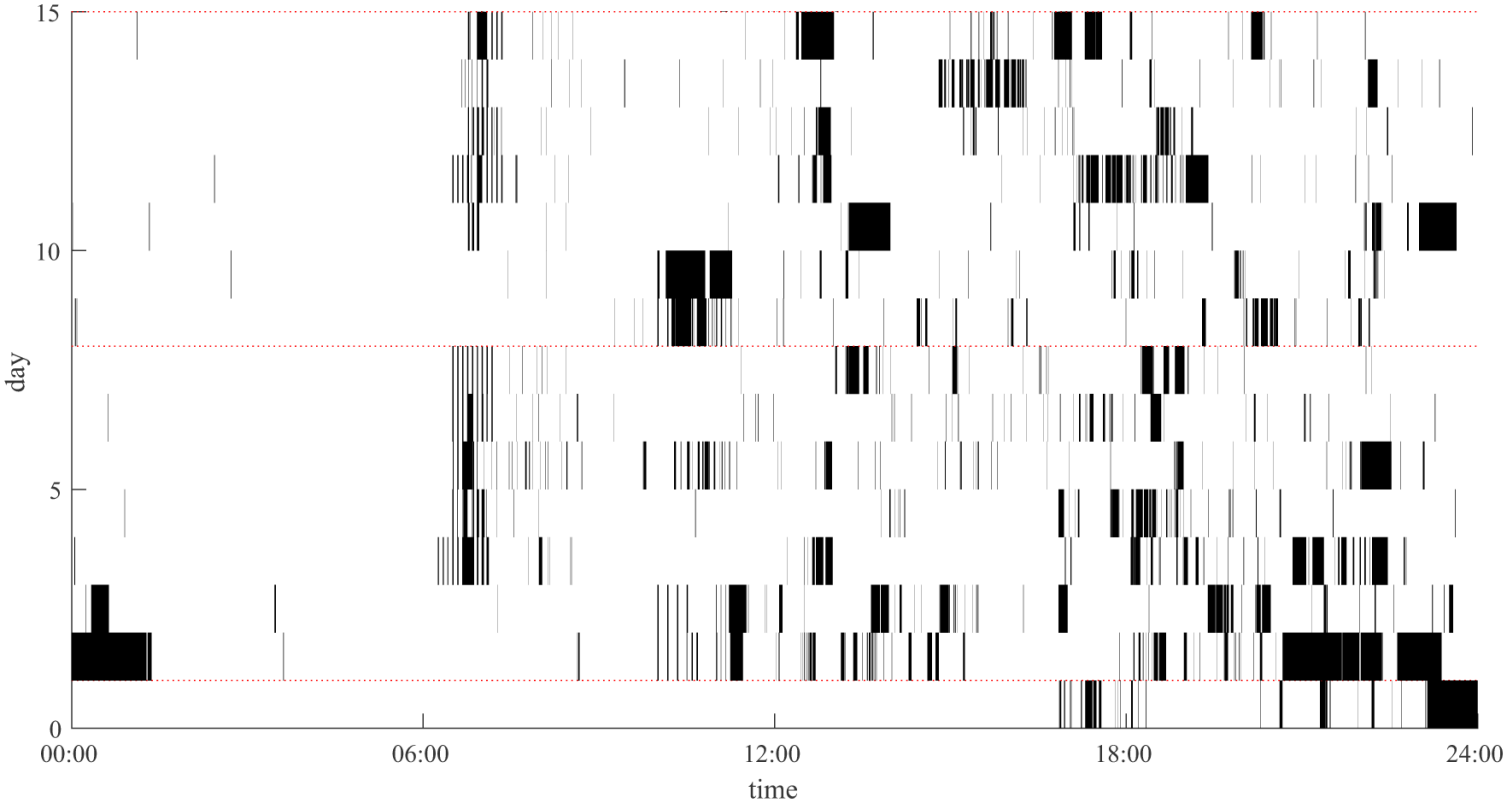
Barcode of smartphone use over two weeks. Black areas indicate times where the phone was in use and Saturdays are indicated with a red dashed line. Weekday alarm clock times (and snoozing) are clearly evident.

Data from the app were converted into a comma separate values file using *Funf* processing scripts. This file was further processed using source code to calculate descriptive statistics and barcode visualisations (as shown in Fig 1; see S1 Appendix for source code). The scripts allow the user to explore different times of day (morning, afternoon, evening, and night), and to explore different metrics associated with checking behaviours of different durations (N.B. the source code requires Matlab 2014b or later). These can be calculated separately for each day, or across the entire duration of a study. We use descriptive statistics for the first 14 days of the study throughout. Some timestamps showed very long single use durations (i.e. > 5 hours). Another limitation to the application is that when the phone switches off, the app does not record the screen turning off. When the phone is turned on again, it also does not record the screen turning on. This results in a seemingly long ‘on’ duration, when the phone itself was actually turned off. It was therefore unclear whether long durations during the day were as a result of the phone being in use (e.g. listening to music, or watching a film), or whether the phone was turned off. As it is impossible to be sure that all long durations were because of this, we retain these data in all analyses, and use median values when calculating an average values for each day, as this is a more accurate summary of the average use length. We then use these values to calculate the mean use length for each participant. We established that occasions when this occurred overnight were the result of the phone being turned off. The included source code (S1 Appendix) enables the visualisation of the data for each participant across all days, or to create an ‘average heatmap’ of one day, seven days, or weekdays and weekends (not shown here).

## Results

### Objective Data

**Fig 2:**
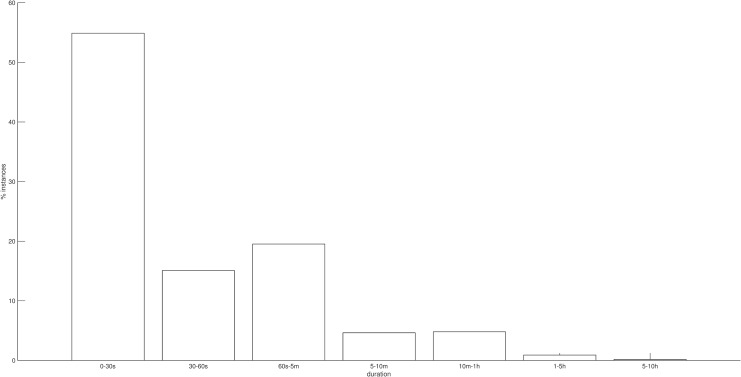
Percentage of uses categorised by duration. This illustrates the highly skewed nature of smartphone usage.

The mean daily number of uses and the mean length of these durations (including a median length for all the durations in a day) and a mean daily duration of phone use (total daily duration) were calculated for each participant. Participants used their phones a mean of 84.68 times each day (SD = 55.23) and spent 5.05 hours each day using their smartphone (SD = 2.73). Length of use was, unsurprisingly, highly skewed, with 55% of all uses less than 30 seconds in duration (see Fig 2).

**Fig 3:**
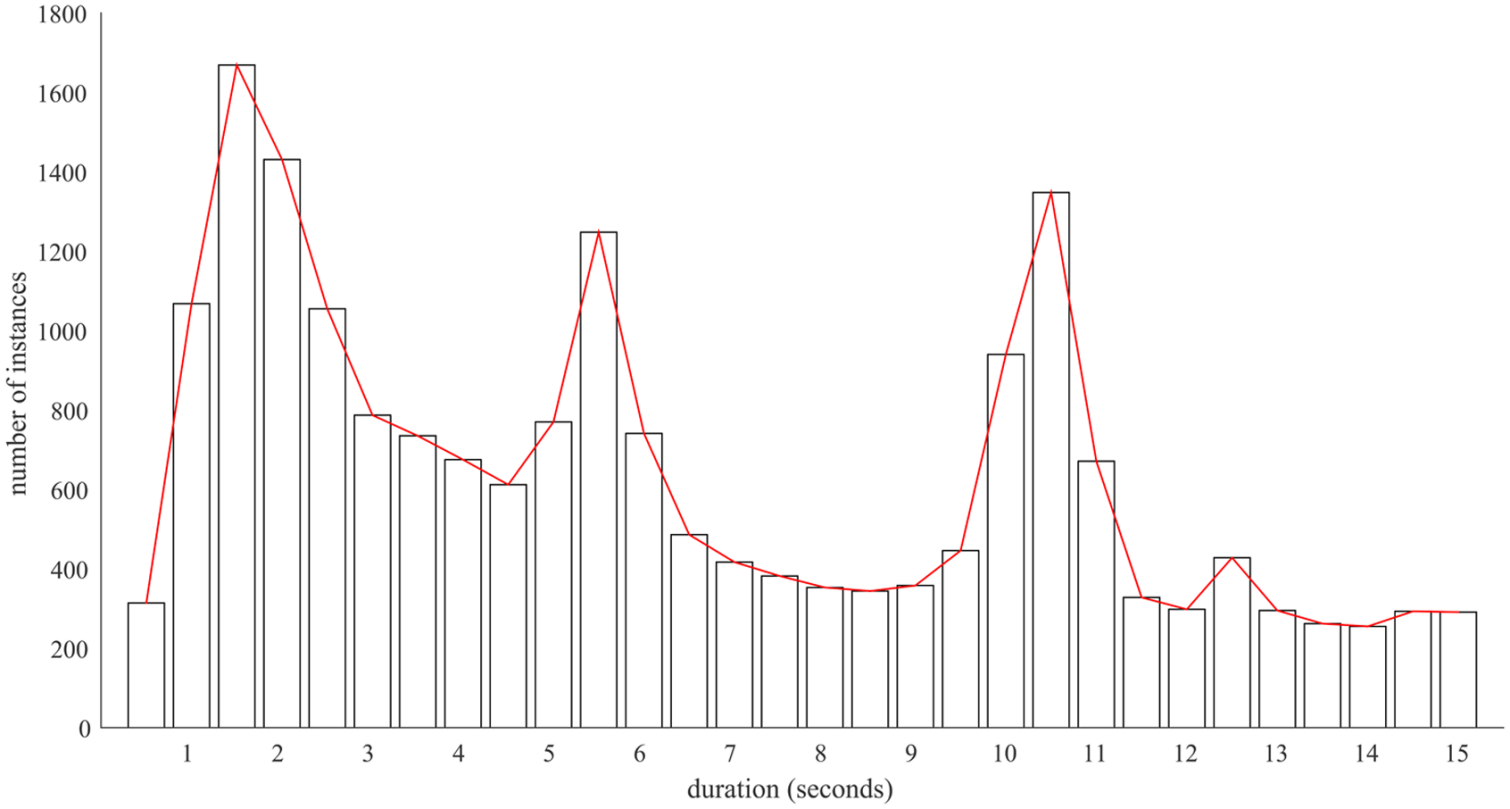
Number of checks in 0.5 second bins across all participants over a 15 second period. Three spikes of checking duration are visible.

We classify ‘checks’ as uses up to 15 seconds in duration. To explore these behaviours more closely, we analysed the percentage of phone interactions with durations under 15 seconds. These showed three distinct periods of increased use; from 1-3s, 5-6s, and 10–11 seconds. Fig 3 shows a histogram of such checks (in 0.5 second bins). In the lab, mean time taken to unlock the phone and read a short message was 8.42s (SD = 1.53). With added distractions outside the lab, the 10-11s time bin is likely to reflect the time taken to read a short message, check the time or other notifications. We explored whether any of these durations could result from the display turning itself off, after a period of being idle. However, results indicated that these default times did not explain any spike in use (default display off times: mean_LOCKED_ = 274.88s, SD_LOCKED_ = 842.85s; mean_UNLOCKED_ = 282.06s, SD_UNLOCKED_ = 524.33s).

**Fig 4:**
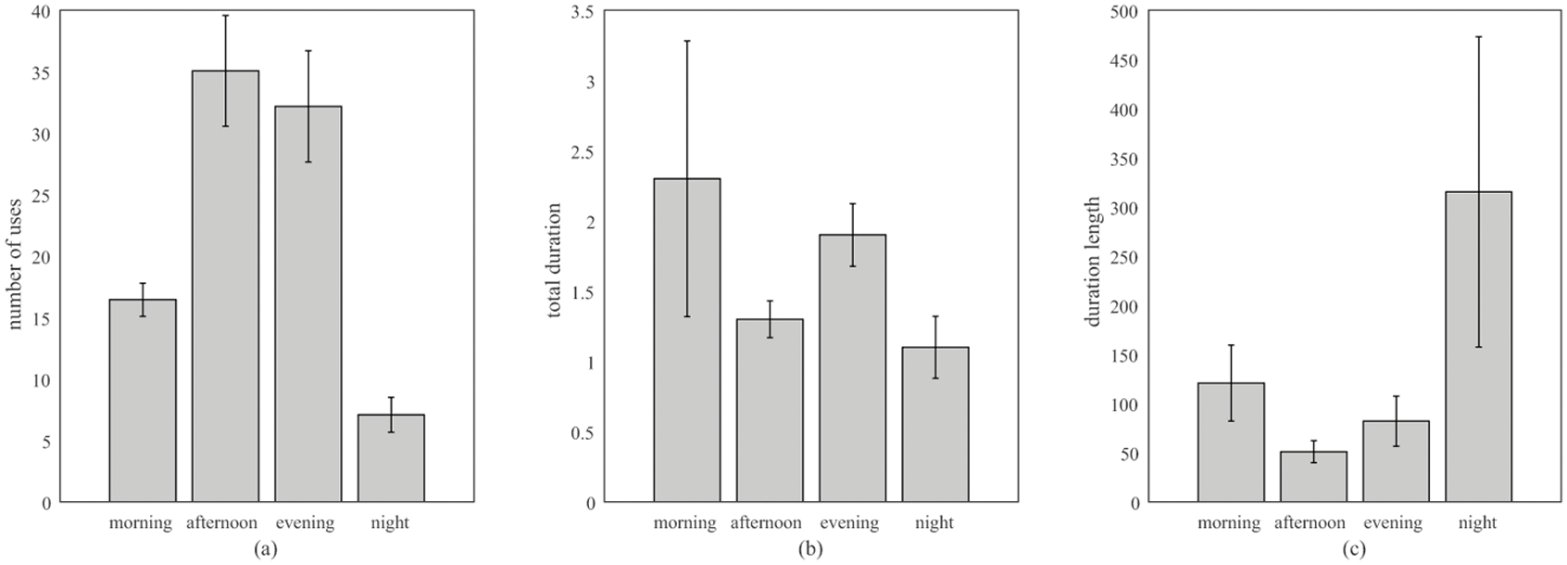
Participants’ mean number of phone uses (a), mean total duration (b), and mean duration length (c) at different times of day. Error bars show 1 SE from the mean.

We also compared phone use at different times of day; night (00:00–06:00), morning (06:00–12:00), afternoon (12:00–18:00), and evening (18:00–24:00), as shown in Fig 4. In this comparison we calculate median duration length—i.e. the median amount of time a user engaged with their phone before turning the display off—for each participant. Finally, we explored the total duration spent using the phone at each time of day. For the purposes of this analysis, phone uses that spanned two time windows (e.g. commencing in the morning and ending in the afternoon) was allocated to the time period in which it originated.

Three one-way repeated measures ANOVAs (Time of Day; morning, afternoon, evening, night) were calculated separately for total daily duration, use length, and number of uses. Data from one participant was removed from total daily duration and median use length analyses, as they had no data from the night time period. There was a significant difference in the number of phone uses at different times of day (*F*(3, 78) = 34.62, *p* < .001, η_ρ_^2^ = .571). Tukey's LSD comparisons revealed more individual uses in the afternoon and evening than in the morning and at night (all *p*s < .001), that there were more uses in the morning than at night (*p* < .001), but that there were no differences in the number of uses between afternoon and evening (*p* = .083). Fig 4a shows these differences. There were no significant differences in total daily duration at different times of day (*F*(3, 78) = .94, *p* = .414, η_ρ_^2^ = .036; see Fig 4b, nor in median use length (*F*(3, 78) = 2.33, *p* = .081, η_ρ_^2^ = .082; see Fig 4c).

### Comparison of objective and subjective measures of smartphone use

We conducted paired-samples t-tests and Pearson correlations to compare actual and estimated smartphone use (see Table 1). For number of phone uses, there were far more actual phone uses (84.68) than were estimated (37.20; *t*(23) = 3.93, *p* < .001), and no significant correlation between the two (*r*(21) = .11, *p* = .610) indicating that estimated number of phone uses does not reflect actual number of uses. For total daily duration there was no significant difference between actual (5.05 hours) and estimated use (4.12 hours; *t*(22) = 1.78, *p* = .086) and there was a moderate positive correlation between the two (*p* = .02). This suggests that estimated duration of use may have reasonable relative validity.

**Table 1:** Correlation matrix of MPPUS scores, and actual and estimated smartphone use.

	Estimated uses	Actual uses	Estimated duration	Actual duration
Actual uses	0.11			
Estimated duration	0.02	-0.03		
Actual duration	0.23	0.12	0.47^a^	
MPPUS	0.03	0.29	0.17	0.30

^a^ *p* = .02

We finally compared scores on the MPPUS with objective and estimated smartphone use and checks using Pearson's correlations (see Table 1). None of these analyses revealed any significant relationships (*p*s > .15). Ten participants scored more than 2SD greater than Bianchi & Phillips’ [14] mean, indicating problem use.

## Discussion

Estimated levels of smartphone use have previously been related to sleep, interpersonal relationships, driving safety, and personality [5, 7, 22, 23]. Here we observe that self-reported estimates of phone use relate moderately to actual behavior in such situations. Conversely, estimated number of checks showed no clear relationship with actual uses; indeed, actual uses amounted to more than double the estimated number. It is possible that our limited sample size obscured a larger effect size. Nevertheless, we suggest that estimated use may not be sufficient if a higher resolution of data are required, but that estimates of total use are likely to be adequate for many research designs. However, for exploring checking behaviours, estimated number of uses show little reliability for measuring actual uses.

The quantity of short checking behaviours we observed are comparable with those found by Oulasvirta and colleagues [24], who collected data in 2009. Smartphone use has become much more prevalent in the intervening six years, and it would be easy to assume that smartphone use would increase accordingly. However, our data indicate that checking behaviours are no more prevalent now than they were six years ago. It is interesting to note that people have little awareness of the frequency with which they check their phone. Oulasvirta and colleagues made this claim in 2012, however this is the first paper to demonstrate that rapid mobile phone interactions are habitual [25]. While phone interactions under thirty seconds have previously been classified as 'checking behaviours', our data suggest that habitual goal-and reward-based actions are likely to be less than 15 seconds in duration when it comes to checking the time or message notifications.

In our study, the MPPUS did not correlate with any measure of phone use—actual or estimated. The MPPUS is used not only as a measure of problem phone use, but also as an additional measure of phone use more generally. To determine validity of the MPPUS for this purpose, we correlated objective phone use with MPPUS scores. This is not to say that the MPPUS lacks validity, but rather that people use smartphones for a variety of reasons [26], and that increased use does not necessitate a problem in itself [27]. It may seem reasonable to assume that those who spend a long time on their phone have problem mobile phone use. However, heavy users are not necessarily the same as problem users. While it is easy to conflate heavy use with problem use, research into smartphone use should identify heavy use and problem use independently of one another (e.g. [8]).

Examining how much people actually use their smartphone can be useful for a variety of applications. For example, all except one of our participants used their phone as an alarm clock, and most reported that they always use their phone last thing before sleeping. These usage patterns therefore provide a non-invasive indication of sleep length, which has the potential to augment sleep diary data [28]. Furthermore, while we have considered usage patterns across the day, a further extension to this analysis would be to consider how these patterns across different days of the week. This is likely to have additional social and occupational consequences [29].

Trull and Ebner-Priemer [9] and Miller [10] argue that smartphone data have a great deal to offer as a research tool in psychology, yet comparatively little research utilises objective smartphone data. Here we show that estimates of smartphone use have a place within current research, but we caution that its validity is limited and should be complimented by measurements of real behaviour. We also provide the first method to automatically sample and easily visualise the frequency of smartphone use with a simple background app. We hope that methods described in this paper will help overcome some barriers to accessing smartphone data for research in psychology and that it will form a foundation to build upon in the coming years.

## Supporting Information

S1 Appendix Source Code for analysing smartphone use data. Source code, example *screenprobe*.*csv* data file, and *README*.*txt* for processing, visualising and analysing smartphone use data. *csv2data*.*m* converts *ScreenProbe*.*csv* to usable data, while *barcode*.*m* allows visualisations to be generated. *descriptives*.*m* generates descriptive statistics that can be used for quantitative analysis. Source code requires Matlab version 2014b or later, but does not require any specific toolboxes.
(ZIP)
